# Communication Barriers in Patient-Provider Interactions in Health Care: Scoping Review

**DOI:** 10.2196/79744

**Published:** 2026-07-21

**Authors:** Moajjem Hossain Chowdhury, Yunmei Liu

**Affiliations:** 1 Department of Industrial and Systems Engineering, University of Louisville, 132 Eastern Parkway, Louisville, KY, United States, 1 502-852-7757

**Keywords:** communication barriers, health care communication, AI in health care, technology intervention, patient-provider communication, artificial intelligence

## Abstract

**Background:**

Effective communication is crucial for high-quality health care, but systemic barriers still disrupt patient-provider interactions. Research shows that communication failures are a major reason for preventable medical errors. These issues are linked to around 30% of malpractice claims and over 1744 deaths each year in the United States. In addition, hospitals lose about US $12 billion each year because of miscommunication. Despite the critical nature of this issue, the literature remains fragmented. Most studies focus on specific communication problems rather than examining how these barriers work together in patient-provider settings.

**Objective:**

This scoping review aims to map the literature thoroughly to (1) identify and categorize key types of communication barriers, (2) evaluate their impacts on patient experience, clinical decision-making, and health outcomes, and (3) examine intervention strategies designed to mitigate these challenges. The findings aim to inform the development of adaptive, technology-enabled solutions to improve patient-provider communication in increasingly diverse and digitally integrated health care settings.

**Methods:**

A scoping review was conducted in accordance with the PRISMA-ScR (Preferred Reporting Items for Systematic Reviews and Meta-Analyses extension for Scoping Reviews) guidelines across four databases: PubMed, IEEE Xplore, CINAHL, and Current Contents Connect. This covered the period from January 1, 2004, to April 30, 2026. The search focused on peer-reviewed, English papers that examined language barriers, cultural barriers, psychological barriers, and mental model differences in clinical settings.

**Results:**

From an initial set of 6233 records, 253 studies met our stringent inclusion criteria. This review divides communication barriers into four categories: linguistic, cultural, psychological, and mental model differences. It highlights four key findings. First, there has been a notable increase in scholarly interest in communication barriers over the last decade, showing greater awareness in clinical fields. Second, although the four barriers have been known, little is known about the specific communication habits or physiological signs defining each barrier. Third, while researchers have linked individual barriers to adverse outcomes, evidence shows that various barriers often happen at the same time. This creates a combined effect that increases patient distress. Fourth, despite the introduction of promising solutions such as interpreter services, cultural training, and AI tools, a lack of basic knowledge about real-time indicators slows the development of intelligent systems.

**Conclusions:**

Unlike previous reviews, which typically examine single communication barriers, this scoping review offers a new approach by mapping how linguistic, cultural, psychological, and cognitive barriers intersect. It provides a unified framework that connects these complex communication failures to specific technological solutions. In practice, this guides the combining of AI tools with real-time physiological monitoring to develop adaptive, patient-centered communication strategies. Ultimately, incorporating these responsive technologies into clinical practice is crucial for lowering medical errors, addressing health disparities, and ensuring fair, patient-centered care.

## Introduction

### Rationale

Communication is the process by which people share information, thoughts, and feelings through verbal, nonverbal, and symbolic methods, all within a social and cultural setting [1]. Effective communication can thus be described as when a communication is understood and acted upon as intended [2]. Hence, effective communication is fundamental to the delivery of quality health care, as it facilitates accurate diagnoses, encourages adherence to treatment plans, and ultimately leads to improved health outcomes for patients [3]. Beyond the exchange of information, communication forms the foundation for developing strong therapeutic relationships, ensuring patient safety, coordinating care, and improving the overall quality of health care [4].

Communication failures, however, remain a pervasive challenge in clinical environments. Communication failure occurs when effective communication cannot occur due to some impediments [5]. These failures have been directly linked to a wide range of adverse outcomes, including misdiagnoses, medication errors, delayed treatments, and compromised patient safety. According to Makary and Daniel [6], among the various reasons for emphasizing effective communication in health care, patient safety stands out as the most critical. Medical errors, having been listed as the third leading cause of death in the United States, often stem from inadequate communication, which plays a significant role in these preventable tragedies [6]. Research shows that over 70% of sentinel events are associated with communication breakdowns, while poor interprofessional communication is linked to more than 60% of all adverse hospital events [7]. The failures can lead to misdiagnoses, incorrect medication administration, procedural mistakes, and delays in delivering appropriate treatment [8].

On the other hand, high-quality communication practices improve the urgency, accuracy, and clarity of clinical interactions, significantly reducing the risk of medical errors [9]. Patients who perceive high-quality communication with health care providers are more likely to adhere to treatment plans, achieve better health outcomes, and express greater satisfaction with their care [10]. Furthermore, hospitals with better patient-reported satisfaction scores, driven largely by communication quality, tend to have lower readmission rates and fewer patient safety incidents [11].

Beyond clinical consequences, communication inefficiencies are associated with substantial financial waste. Based on Agarwal et al [12], in the United States, hospitals collectively waste over $12 billion annually due to communication breakdowns among health care providers, with increased patient length of stay accounting for 53% of this burden. For instance, a 500-bed hospital may incur an annual loss exceeding $4 million because of these inefficiencies, equating to about 2% of total hospital revenue nationwide [12]. Furthermore, communication failures contributed to 30% of medical malpractice claims from 2009 to 2013, leading to 1744 fatalities and $1.7 billion in legal settlements [13].

Despite its critical importance, communication in health care remains a complex and multifaceted process. It occurs not only between providers and patients but also among interdisciplinary teams and across institutional boundaries. These interactions are often shaped by factors such as organizational structure, time constraints, cultural differences, and technological systems [4]. As health care continues to evolve toward value-based care models and increased emphasis on patient outcomes, the role of effective communication becomes even more critical for achieving the goals of improved patient safety, enhanced care coordination, and better health outcomes [7].

Having established that communication breakdowns carry measurable human, economic, and safety costs, moving from why communication matters to what specifically impedes it is essential. Literature consistently converges on four recurrent obstacle classes: language discordance, cultural incongruence, psychological distress, and mismatched mental models, that operate at different yet interconnected levels of the care encounter. In this review, we define these four classes a priori, drawing on existing theoretical and empirical literature. We believe each class has sufficient grounding to guide our coding and analysis. The definitions are summarized in Multimedia Appendix 1, and they provide the conceptual basis for interpreting the empirical patterns examined in this review.

Language-based impediments emerge whenever patients and clinicians lack a shared linguistic repertoire or a common foundation of health literacy. Limited English proficiency (LEP), dependence on ad hoc interpreters, and the routine use of jargon that exceeds a lay audience’s comprehension all constrain the accurate encoding, transmission, and decoding of clinical information, thereby jeopardizing diagnostic precision and safe decision-making [14,15]. Prior studies confirm this impact. Al Shamsi et al [16] report that language barriers are linked to poorer outcomes and trust. Gerchow et al [17] highlight how language differences disrupt nurse-patient communication and weaken therapeutic relationships. Meuter et al [18] show that language discrepancies add psychological stress and increase the risk of clinical errors.

Cultural barriers are rooted in divergent belief systems, values, social norms, and religious prescriptions that shape how illness is conceptualized, how authority is negotiated, and which therapeutic options are deemed acceptable [19,20]. When patients prioritize traditional remedies, observe gender-specific interaction rules, or interpret bodily symptoms through culturally embedded explanatory models, biomedical discourse can appear alien or even threatening, eroding mutual trust [21,22]. Conversely, clinicians untrained in cultural humility may unintentionally impose ethnocentric assumptions, exacerbating miscommunication and widening disparities [23]. Betancourt et al [24] argue that cultural beliefs and expectations, when not addressed, act as barriers to effective care. Vandecasteele et al [25] find that general practitioners often struggle to meet the needs of patients from different cultural backgrounds.

Psychological barriers arise from intrapsychic states, stigma, fear, anxiety, embarrassment, or emotional overload that limit a patient’s willingness or ability to disclose sensitive information, ask clarifying questions, or participate in shared decision-making [26-28]. Anticipation of judgment, particularly in mental health or stigmatized conditions, can prompt reticence or care avoidance, delaying diagnosis and compromising outcomes [29,30]. Even in nonpsychiatric contexts, acute stress narrows cognitive bandwidth and impairs information retention, reducing the effectiveness of risk communication and consent processes [31]. Gheshlagh et al [32] identified psychological distress as a key barrier in nurse-patient interactions. Meuter et al [18] also show that language discrepancies can heighten distress, making communication even harder.

Finally, mental model difference (MMD) refers to cognitive incongruities between patients and health care professionals that stem from disparate educational backgrounds, experiential histories, and cultural frames of reference [33,34]. While clinicians interpret illness through evidence-based taxonomies and probabilistic reasoning, patients frequently rely on analogical or culturally derived schemas that privilege immediate symptom relief or lay etiologies [35,36]. Such misalignment destabilizes shared understanding, hampers adherence, and diminishes the efficacy of decision aids to support collaborative planning [33]. Without explicit metacommunication to surface and reconcile these implicit frameworks, both parties risk talking past one another, undermining the goals of patient-centered care. Even when language is shared, such mismatches create misunderstandings. Kwame and Petrucka [2] describe a “communication chasm” in patient-centered care that emerges when patients interpret nonverbal cues or medical reasoning differently than providers. Partida [37] adds that people attach different meanings to the same medical terms based on personal or cultural background, which further destabilizes shared understanding.

Despite significant advancements, a critical gap remains in our understanding of how various communication barriers interact and collectively affect patient outcomes and health care delivery. Most existing studies tend to focus on isolated barriers or specific interventions, often neglecting the complex interplay of multiple factors that shape communication challenges across diverse health care settings [16,18,38-40]. For instance, while linguistic barriers are widely acknowledged [16,18,38,39], there is less emphasis on how factors such as psychological distress, cognitive incongruities, and cultural misunderstandings can exacerbate communication difficulties. Similarly, while AI-powered tools hold great potential for addressing language barriers, their effectiveness may be compromised in emotionally charged interactions where empathy and human connection are vital [41-43]. Given these complexities, there remains a pressing need for systematic reviews that take a comprehensive approach to analyzing communication barriers in patient-provider settings.

### Objectives

The objectives of this study include the following: (1) to identify the most commonly discussed communication barriers; (2) to identify their impacts on patient outcomes and health care delivery; and (3) to explore interventions designed to address these barriers and which barrier types they primarily target.

The rest of this paper is organized into the following sections: the Methods section describes the research methodology, the Results section presents the results, and the Discussion section analyzes the results and discusses their implications.

## Methods

### Protocol and Registration

This section outlines the methodological approach used to conduct the scoping review. This study was not registered.

### Eligibility Criteria

To ensure consistency in including relevant literature, the eligibility criteria for this scoping review were based on the Population, Concept, and Context framework. This review included studies that involved both patients and health care providers, such as physicians and nurses, engaging in clinical interactions. Studies were excluded if they did not clearly focus on the patient-provider relationship. The main concept of interest was communication barriers in health care. To be included, studies had to examine one or more of the following:

- The identification and categorization of communication barriers, specifically linguistic, cultural, psychological, and MMDs.
- The impact of these communication barriers on patient outcomes, patient experience, or clinical decision-making.
- Interventions, tools, or strategies, including technological solutions, designed to reduce these barriers.

The context for this review included any formal health care setting. This involved acute care hospitals, primary care clinics, emergency departments, and telehealth environments. Studies conducted in non–health care settings were excluded. This review looked at primary empirical studies of any design, including quantitative, qualitative, and mixed-methods research. To ensure the synthesis was based on high-quality, current evidence, inclusion was limited to peer-reviewed journal papers published in English between January 1, 2004, and April 30, 2026. Opinion pieces, editorials, and commentaries without empirical data, as well as secondary research such as previous systematic or scoping reviews, were excluded.

### Information Sources

To strengthen the transparency and reproducibility of our literature search, we report our methodology in accordance with the PRISMA-S (Preferred Reporting Items for Systematic Reviews and Meta-Analyses literature search extension) guidelines [44]. A comprehensive, sequential literature search was conducted across four electronic databases: PubMed, IEEE Xplore, CINAHL, and Current Contents Connect. The paper search was completed on May 4, 2026.

### Search

The search strings were constructed iteratively by the research team using Boolean operators to combine key concepts surrounding communication barriers, health care settings, and patient outcomes. The complete database-specific search histories, including the exact search strings and applied filters for PubMed, IEEE Xplore, CINAHL, and Current Contents Connect, are provided in Multimedia Appendix 2. These search histories were copied directly from the respective database search-history records and are presented in Tables S1-S4 to support transparency and reproducibility. The searches were limited to papers published between January 1, 2004, and April 30, 2026. In accordance with our scoping review methodology, we did not search clinical trial registries, unindexed online search engines, or gray literature databases. Furthermore, neither did we conduct manual handsearching of specific journals, forward or backward citation searching (snowballing) of included papers, nor did we contact study authors to identify additional or unpublished sources of evidence.

### Selection of Sources of Evidence

Following the execution of the search, which yielded 7756 initial papers, all identified records were exported and managed using Rayyan AI, a web-based systematic review software [45]. Exact duplicate records were identified and removed by the software, leaving 6233 papers for further screening. The study selection process now involves two stages of screening:

- Title and abstract screening: studies were initially screened for relevance based on their titles and abstracts.
- Full-text review: full texts of potentially eligible studies were retrieved and reviewed for final inclusion.

### Data Charting Process

In an in-depth review of the selected studies, we extracted all available information on communication barriers, their impact on patient outcomes, and the interventions used to address them. The data collection focused on identifying linguistic, cultural, psychological, and cognitive barriers, as well as technological constraints and systemic challenges in health care communication. These extracted notes form the basis of the following review, providing a structured synthesis of recurring challenges, intervention strategies, and their effectiveness across diverse health care settings.

### Data Items

For each included source of evidence, the following variables were charted: bibliographic details (author, year, and country of data collection); participants and outcomes; the specific type or types of communication barrier examined (linguistic, cultural, psychological, MMDs, or combinations thereof); reported impacts of the barrier on patient experience, clinical decision-making, health care access, or health outcomes; and any interventions, tools, or strategies evaluated to mitigate the identified barriers.

### Critical Appraisal of Individual Sources of Evidence

Consistent with established scoping review methodology, a formal critical appraisal of the methodological quality or risk of bias of individual sources of evidence was not undertaken. The purpose of this review was to map the breadth and range of the available literature on communication barriers in patient-provider interactions rather than to evaluate the strength of evidence supporting specific interventions or outcomes.

### Synthesis of Results

The charted data were synthesized using a descriptive, narrative approach supplemented by quantitative summaries of publication trends and visual mapping. Studies were grouped by barrier type (linguistic, cultural, psychological, and MMDs) and by impact domain (patient experience, health care access, clinical decision-making, health outcomes, and system efficiency). Publication frequency over time was summarized graphically to characterize the temporal evolution of the field, and the intersection of barriers and impact domains was visualized using an evidence map. Findings are presented in tabular and narrative form, organized to address each of the review research questions.

## Results

### Selection of Sources of Evidence

**Figure 1.**
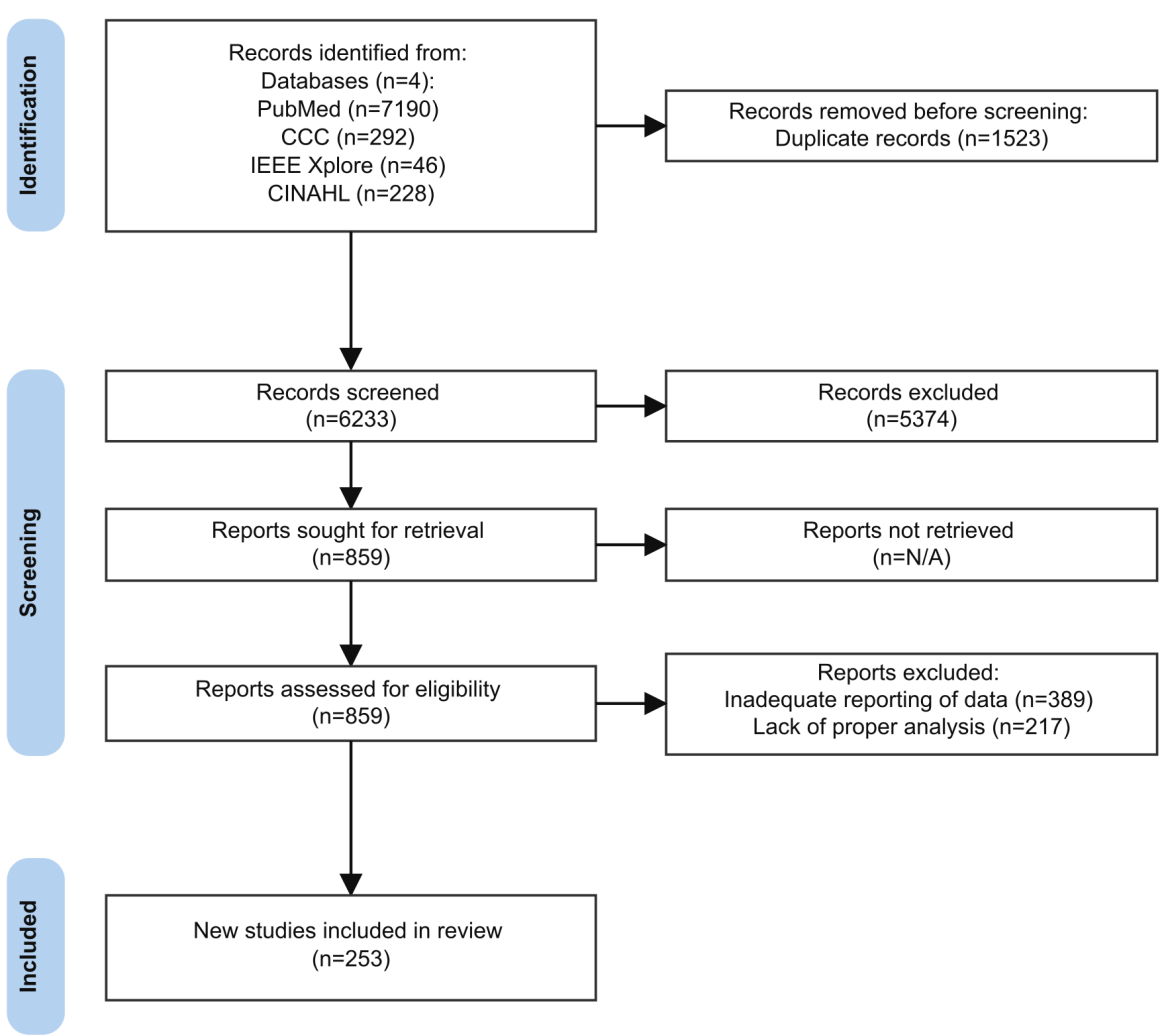
This diagram illustrates the systematic screening process of 253 empirical studies examining communication barriers between patients and health care providers worldwide. The search was conducted across four databases (PubMed, IEEE Xplore, CINAHL, and Current Contents Connect) and encompasses peer-reviewed literature published between January 2004 and April 2026. N/A: not applicable.

Figure 1 depicts the screening process, which was conducted using Rayyan AI [45]. Records were eliminated due to duplication, abstract and title review, and full-text screening. This resulted in 253 papers to be reviewed from 6233 papers screened.

### Characteristics of Sources of Evidence

**Figure 2.**
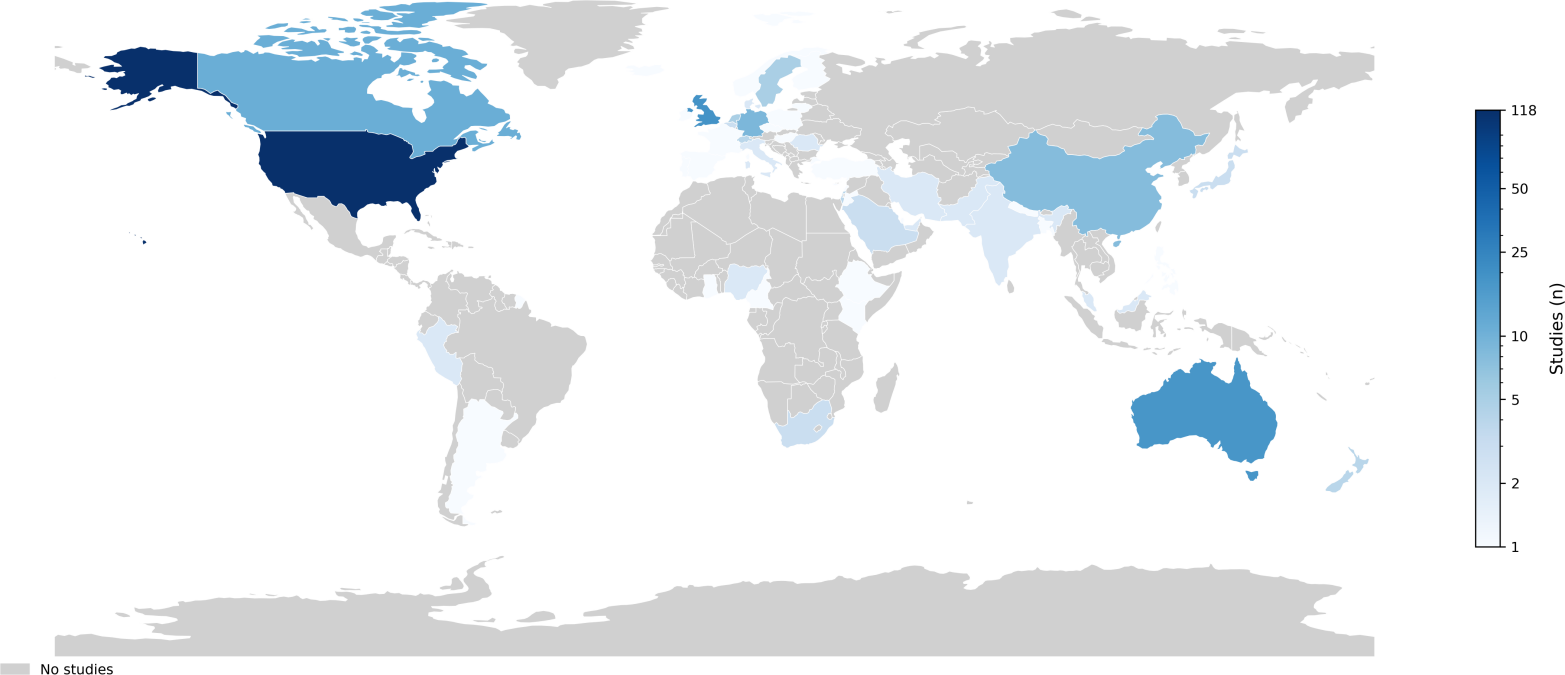
Geographic distribution of included studies by country of origin (N=253). Countries are shaded according to the number of studies conducted within their health care settings, using a logarithmic color scale. Gray indicates no studies.

The geographic distribution of included studies (Figure 2) indicates a pronounced concentration of evidence in high-income, English-speaking settings. The United States accounted for nearly half of all included studies (n=118), followed by the United Kingdom (n=19) and Australia (n=18). Although the evidence base included studies from 47 countries across Europe, East Asia, Africa, and the Middle East, several regions remained substantially underrepresented, particularly sub-Saharan Africa, Latin America, and Central Asia, where most countries contributed only one study. This geographic imbalance suggests that the literature on communication barriers in patient-provider interactions is shaped largely by research conducted in multicultural, high-income health care systems. Such settings may have greater visibility of language, cultural, and health-literacy challenges because they serve highly diverse patient populations.

**Figure 3.**
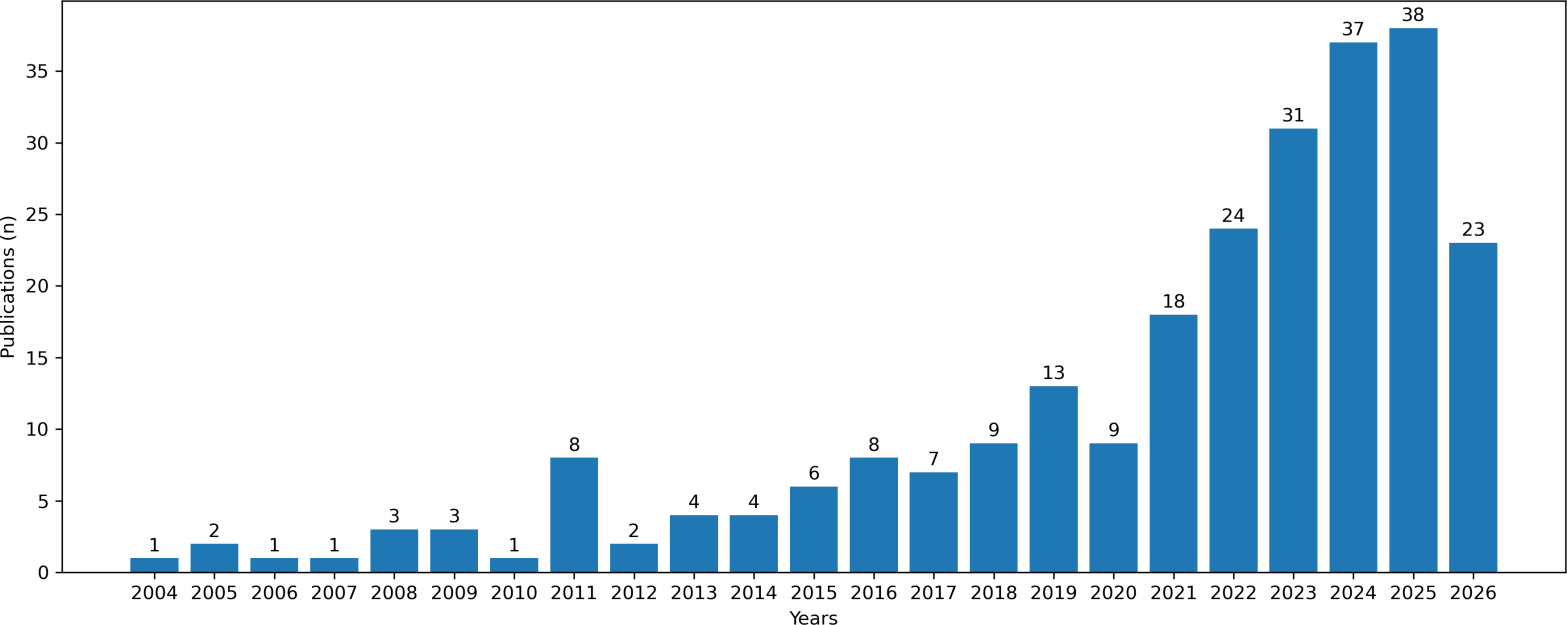
The bar chart illustrates the annual publication volume of the 253 studies included in this scoping review from 2004 to April 2026. The data demonstrate a marked acceleration in scholarly activity related to patient-provider communication breakdowns starting in 2019, reflecting a growing global focus on clinical communication in diverse health care settings.

Figure 3 illustrates the annual publication output on communication barriers in health care from 2004 to 2026, revealing a clear three-phase trajectory in scholarly activity. Phase 1 (2004‐2009) is characterized by an almost complete absence of relevant papers, underscoring the field’s embryonic status and minimal academic attention to the topic. Phase 2 (2010‐2018) shows a modest but inconsistent increase in publication volume. During this period, annual output remained at or below 7 papers, apart from a spike to 10 papers in 2018. This pattern suggests the topic remained niche, with limited but emerging academic exploration. Phase 3 (2019‐2026) marks a pronounced inflection point characterized by sustained and accelerating growth. This represents the field’s transition from exploratory inquiry to rapid thematic consolidation and rapid expansion.

**Figure 4.**
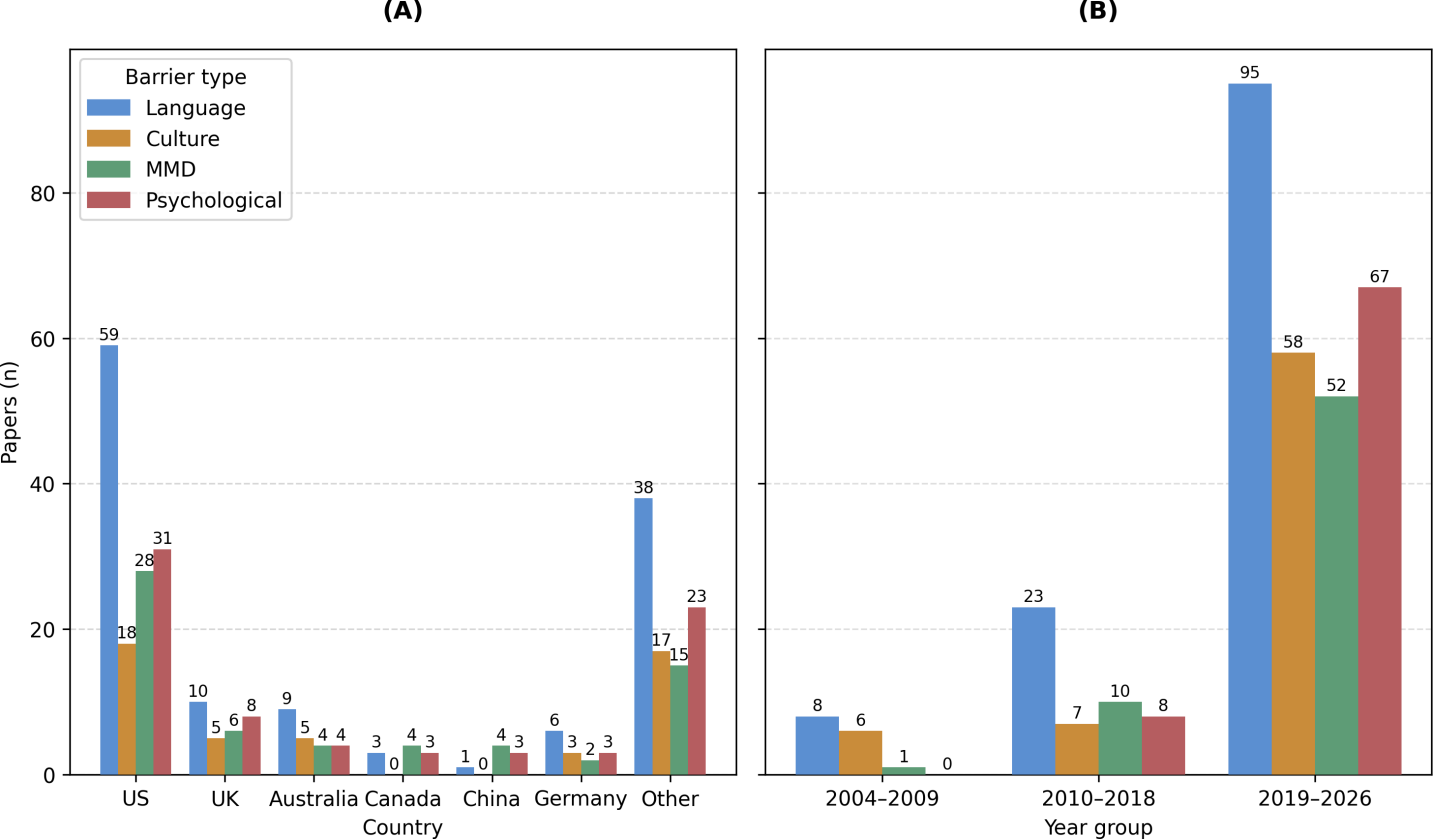
Distribution of included papers by barrier type across countries and publication periods. The left panel shows the number of papers addressing language, cultural, MMD, and psychological barriers in the six most frequently represented countries. The right panel shows the temporal distribution of studies across three publication periods. Language barriers were the most frequently reported barrier type across both country-level and year-group distributions, with a marked increase in studies published during 2019‐2026. Note that 2026 contains publications till April. MMD: mental model difference.

Based on the geographical information from Figure 2, Figure 4A shows the breakdown of the six most frequently represented countries. The figure shows that the country-level evidence is concentrated primarily in the United States, where all four barrier types were reported more frequently than in any other country. Language barriers were the dominant category in the United States (n=59), followed by psychological barriers (n=31), MMD barriers (n=28), and cultural barriers (n=18). In comparison, the United Kingdom, Australia, Canada, Germany, and China contributed substantially fewer studies across all barrier types. Among these countries (except for China), language barriers generally remained the most frequently reported category. China, as a country with a largely homogeneous culture, had no study of cultural barriers, with studies in China mainly focusing on MMD and psychological barriers.

Based on the temporal phases identified in Figure 3, Figure 4B shows the distribution of the number of papers by barrier type across three phases. The embryonic nature of the study is once again shown here, as the group with 2004 to 2009 has fewer than 20 studies in total. In general, we see increasing numbers of publications as years pass in all barrier types. When examining barrier types, it is clear that the significant emphasis on language-related issues reflects a historical tendency in the literature to view miscommunication mainly as a linguistic challenge. In contrast, there has been less systematic focus on cognitive misalignment or patients’ emotional states. While cultural factors receive moderate coverage, reflecting their recognized influence on health discourse, the comparatively sparse treatment of mental-model and psychological barriers suggests two underexplored avenues.

**Figure 5.**
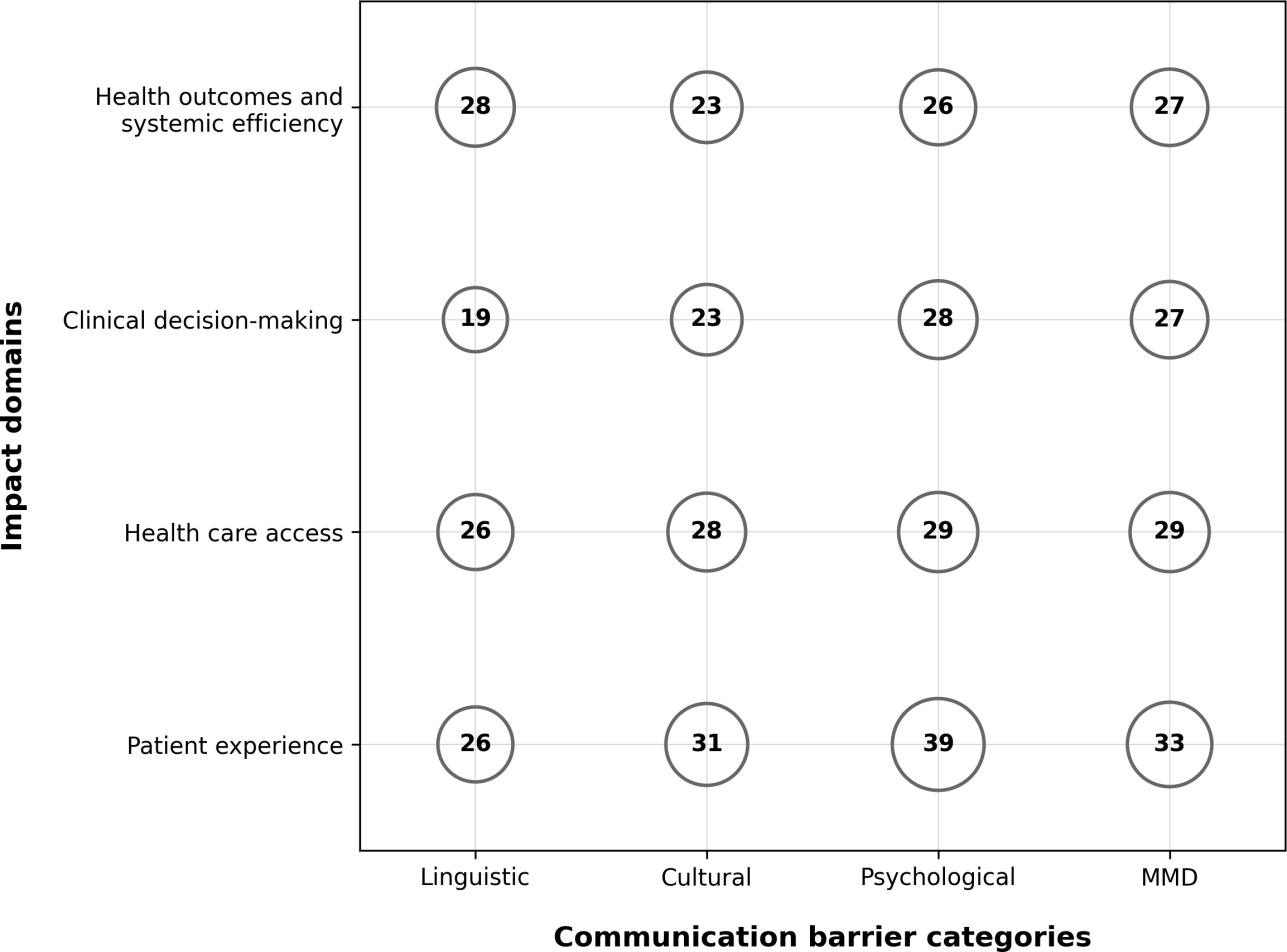
Evidence map of research density intersecting communication barriers and clinical impact domains. This bubble chart synthesizes findings from 253 empirical studies (January 2004 to April 2026). Bubble size corresponds to the relative frequency of citations identified in the scoping review. MMD: mental model difference.

A bubble chart is depicted in Figure 5, where communication barriers and their impact domains are mapped together. That is to say, the chart shows how each barrier affects health outcomes, clinical decision making, health care access, and patient experience. The numbers represent the number of papers that studied the barrier and discussed the impact. It can be seen that impact domains across the barriers are quite evenly studied.

### Synthesis of Results

#### Overview

The current literature commonly discusses four distinct communication barriers: language, cultural, psychological, and MMDs. They form the analytical backbone of this review and are classified and further explored in this subsection. Each barrier is examined in turn, with attention to its defining characteristics and the clinical context in which it most commonly manifests.

#### Language Barriers

Language barriers between patients and clinicians remain a foundational threat to safe and effective care (Table 1). When the clinician and patient do not share a common tongue, the probability of diagnostic inaccuracies, medication errors, and diminished therapeutic alliance rises sharply [46-57]. Even among bilingual encounters, nuanced clinical terminology can be lost, and patients often resort to a rote agreement rather than clarifying doubts, thereby masking unresolved concerns [53-59]. This dynamic becomes more complicated due to systemic factors. Research shows that LEP mothers often feel abandoned by the health care system. They deal with long wait times and staff who lack cultural understanding while trying to navigate complex institutions to obtain diagnoses and services for their children [60]. Studies of nonnative English speakers further show that LEP systematically reduces comprehension of illness trajectories and adherence to treatment recommendations, ultimately widening health-outcome disparities [50,61-68]. This situation makes patients feel powerless. In adult acute care settings, patients often mention language barriers as their main frustration. They point out that ignoring cultural customs can create feelings of racism and exclusion [69].

**Table 1.** Based on the scoping review of empirical literature, this table synthesizes key language-based barriers to care. It identifies how LEP^a^, reliance on untrained ad hoc interpreters, and professional interpreter shortages disrupt clinical communication for diverse patient populations across global health care settings.

Context	Barrier	References
Patients and providers do not speak the same language, leading to miscommunication and medical errors.	Language mismatch	[46-64,69-142]
LEP hinders understanding of diagnosis and treatment, especially in nonnative speakers.	LEP communication	[50,60-69,78,83,90,91,97,107,110,116-132,134,136-138,141-153]
Dependence on family members or untrained interpreters increases the risk of misinformation.	Untrained interpreter use	[51,53,109,118-123,126,128,131,132,137-139,146,150,154-163]
A shortage of professional interpreters affects care quality in emergencies and rural settings.	Interpreter shortage	[109,118,119,121,126,128,132,138,146,161-171]

a LEP: limited English proficiency.

In the absence of qualified language services, many encounter default to ad hoc solutions—most commonly family members, untrained staff, or informal community volunteers. Such substitutions increase the risk of omitted symptoms, distorted risk explanations, and breaches of confidentiality [51,53,109,146,154,156,160-162]. In neonatal care, informal interpretation by multilingual health care staff shows limited formal support. While empirical analyses of emergency departments and rural clinics show that untrained interpreters nearly double error rates in medication reconciliation and consent [150,155,157,163]. The serious effects of these communication gaps were clearly shown during the COVID-19 pandemic. Communication problems in critical care, worsened by isolation and mechanical ventilation, required simple, low-tech teamwork between nurses and speech-language pathologists to ensure basic communication between patients and providers [75].

Compounding the problem, many regions face chronic shortages of professional interpreters, a gap that becomes acutely visible during off-hours and high-acuity scenarios [146,161,164,165,167]. As health care shifts more to digital platforms, older adults with language barriers face a double challenge. They deal with both language issues and technology exclusion, making it hard to connect with care teams through telehealth services [80]. These shortages undermine quality-of-care metrics and intensify clinician stress, especially when rapid, high-stakes decision-making is required [146,161,163,168,169].

#### Cultural Barriers

Beyond language, cultural frames influence how information is delivered, interpreted, and acted upon (Table 2). Conflicting belief systems regarding disease etiology, family authority, and preferred healing practices can derail shared decision-making unless clinicians actively negotiate meaning [53,63,105,109,116,172-175]. Menopausal women may avoid discussing sexual health because they believe that unmarried or younger providers cannot understand their traditional marital duties [86,176]. In another case, older adult Arab patients felt that their cultural and religious beliefs were not understood, which led to health services being seen as less effective [123]. Similarly, patients with breast cancer identified ingrained cultural beliefs as a key barrier to discussing sexual health [177]. Even ostensibly simple nonverbal cues, such as eye contact duration, tone, or gestures, carry divergent connotations across cultures, sometimes signaling disrespect when none is intended [52,109,178,179]. This challenge increased during the COVID-19 pandemic, when personal protective equipment obscured facial expressions. Clinicians had to depend on basic “proxy” methods such as shouting or using gestures, often weakening rapport [85]. Additionally, the quick move to audio-only telehealth has further damaged these nonverbal connections. This change has hit vulnerable patients hard, as they often need visual cues to understand empathy and trust [91]. Furthermore, norms around disclosure and deference to medical authority differ markedly; some patient communities expect paternalistic guidance, whereas others prioritize egalitarian dialogue [51,180-184]. In acute care, patients with limited English skills often feel “powerlessness” when providers overlook cultural practices, such as family-centered decision-making. The neglect is often interpreted as racism [69]. Effective engagement with immigrant communities often requires trusted faith-based or community networks rather than reliance on traditional clinical authority alone [98,184,185]. Unless these implicit rules are surfaced and reconciled, misalignments in expectations and decision roles persist, thereby perpetuating treatment delays and dissatisfaction [186,187]. Similarly, the rapid move to audio-only telehealth has damaged nonverbal connections, disproportionately affecting vulnerable patients who rely on visual cues to gauge empathy and establish trust [51,184,185].

**Table 2.** Categorization of cultural barriers affecting clinical interactions. Key barriers range from conflicting health belief systems and nonverbal misinterpretations to divergent norms regarding medical authority and diagnostic disclosure.

Context	Barrier	References
Cultural values and norms create misunderstandings in diagnosis and treatment planning.	Belief system conflict	[49,52,53,63,86,98,105,108,109,116,122,123,125,127,132,133,144,172-177,188-193]
Patients from different cultural backgrounds may misinterpret nonverbal cues or communication styles.	Nonverbal communication	[51,52,54,62,63,85,91,109,118,120,122,125,129,132,137,143,151,171,178,179,194-199]
Communication in multicultural settings is complicated by conflicting norms around authority and disclosure.	Authority and disclosure	[51,69,86,101,118,119,122,123,125,126,180-187,191,192,200-203]

#### Psychological Barriers

Fear and anxiety emerge as potent inhibitors of patient‐initiated dialogue during clinical encounters (Table 3). This leads to patients avoiding questions, underreporting pain, or withholding discomfort details, thus compromising diagnostic accuracy and shared decision-making [63,114,204-206]. This reluctance is particularly strong among vulnerable groups. For instance, Brazilian immigrants, whose mistrust of authority can limit health service engagement [98]. Qualitative observations in oncology and surgical settings show that heightened procedural anxiety fuels silence, which in turn amplifies uncertainty and stress, ultimately reinforcing the original fear [207]. These findings underscore the need for anticipatory counseling and structured question prompts that normalize patient participation before anxiety has a chance to escalate.

**Table 3.** Classification of psychological barriers inhibiting patient engagement. It details how acute fear, mental health stigma, emotional overload, and a lack of psychological safety prevent patient disclosure and engagement across various clinical environments.

Context	Barrier	References
Fear and anxiety during treatment make patients hesitant to ask questions or express discomfort.	Fear and anxiety	[63,98,104,111,114,117-119,121,126,191,196,201,204-213]
Stigma about mental illness discourages patients from disclosing emotional or psychiatric issues.	Mental health stigma	[60,63,84,111,117,176,187,192,214-217]
Patients under emotional distress are less likely to articulate concerns clearly, especially during crises.	Emotional overload	[75,84,88,118,122,132,169,176,177,191,194,213,218-223]
Negative emotions such as embarrassment, fear of judgment, or low confidence reduce communication.	Emotional discomfort	[104,111,119,121,126,128,132,139,151,191,192,196,197,201,209,213,222,224-226]
Psychological insecurity and lack of psychological safety undermine trust and hinder communication.	Psychological safety	[86,114,118,119,122,125,126,132,133,135-139,143,151,152,186,191,196-199,201-203,209,211,213,227-235]

The stigma surrounding mental illness compounds this communicative reticence. Patients who internalize cultural or societal prejudice against psychiatric disorders frequently mask depressive symptoms, avoid admitting substance-use histories, or downplay stress levels, thereby limiting clinicians’ ability to form holistic treatment plans [63,111,117,187,214]. For mothers of children with autism and limited English skills, this stigma can compound feelings of abandonment and limited access to support networks [60]. Likewise, during the Ukrainian refugee crisis, stigma and low mental health awareness kept traumatized people from obtaining the available psychosocial support [84].

The effect intensifies under emotional overload, acute periods of crisis, or high-stress hospitalization, when cognitive bandwidth narrows, and expressive capacities falter [169]. This was clearly shown during the COVID-19 pandemic, when critically ill patients on mechanical ventilation experienced “extreme frustration and fright.” They needed simple, low-tech solutions from speech pathologists just to express their basic needs [75,221,222]. In less urgent but still significant situations, low-income women with gestational diabetes found information from their providers to be “overwhelming.” Anger is also seen in a study of 22 Israeli oncologists [220] where patients with cancer and their family are unable to deal with the realities of cancer treatments. This led to a breakdown in communication, as they struggled to process actionable advice because of the overwhelming number of medical instructions [219]. Emergency department interviews demonstrate that distressed patients offer disjointed narratives, omit chronological details, and struggle to prioritize symptoms, leading to mistreated acuity and longer lengths of stay [194,218]. Interventions that combine destigmatizing language with phased, guided questioning have shown promise in recovering essential information under these conditions.

Even outside overt crises, embarrassment, fear of judgment, or low self-confidence consistently predict reduced communication quality. This leads to shorter responses and the use of more hedging phrases, both of which clinicians can misinterpret as disinterest [104,111,224,225]. Concurrently, a lack of psychological safety, such as perceived permission to speak candidly without ridicule or dismissal, erodes trust and discourages disclosure of sensitive issues such as nonadherence or complementary-medicine use [143,209,227]. Case-control data from primary-care clinics indicate that establishing clear “permission statements” (“many patients find this topic awkward…”) nearly doubles rates of honest reporting [186,226].

#### MMDs

Gaps between clinicians’ diagnostic logic and patients’ lay reasoning erode trust and foster decisional paralysis (Table 4). When pathophysiology is explained through jargon-laden narratives or abstract probabilities, patients often fail to grasp causal links between test results and prescribed regimens, leading to confusion, lowered adherence, and a perception that decisions are opaque or arbitrary [111,206,220,227,236]. Patients in acute care often express a feeling of “powerlessness” when complex medical explanations cause them to feel left out of the decisions that affect their health outcomes [69]. These “reasoning gaps” encourage patients to seek confirmatory information from less reliable sources, which can weaken subsequent consultations [143,237,238]. In telephone-only telehealth, the loss of visual cues makes it harder for clinicians to confirm understanding [91]. Explicitly mapping diagnostic steps with plain-language analogies or decision aids has been shown to restore shared understanding and rebuild confidence in the clinical process [239].

**Table 4.** Breakdown of mental model differences between patients and health care providers. Synthesizing findings from the scoping review, this table highlights cognitive discrepancies that disrupt clinical interactions. It specifically details misalignments in diagnostic reasoning, divergent assumptions regarding disease etiology, and conflicting treatment timeline expectations between clinical staff and patient populations.

Context	Barrier	References
Patients struggle to understand medical reasoning or diagnostic logic, leading to confusion and loss of trust.	Reasoning gap	[69,77,83,91,111,118,120-122,125,127-129,132,136,137,143,151,153,176,191,196,206,212,220,227,232-234,236-243]
Differing assumptions about the causes of illness or treatment responsibilities result in breakdowns in decision-making.	Assumption divergence	[63,111,118,122,125,127,132,133,168,192,196,197,200,202,206,210,220,232,237-239,242,244-248]
Patients’ expectations about quick symptom relief or immediate outcomes conflict with providers’ long-term care plans.	Expectation conflict	[33,36,63,77,83,111,122,126,127,132,137,139,151,176,177,192-194,196-198,200,201,203,206,207,210,213,220,232,245,247,249]

Misaligned assumptions about disease etiology and responsibility for follow-through create a second layer of mismatch. Patients may emphasize social stressors or spiritual causes, while clinicians foreground biomedical mechanisms. Similarly, providers may assume that patients will self-monitor symptoms or adjust lifestyle factors, whereas patients expect directive, clinician-led management [168,200,206,220,244]. Patients often have differing assumptions about technology use and the roles of providers [111,245]. Ethnographic data demonstrate that these divergent premises generate conflicting action plans, stalled referrals, and breakdowns in medication titration [237,244]. Structured elicitation of patients’ causal beliefs has been correlated with higher concordance in goal setting and a measurable reduction in follow-up ambiguities [238,239].

Expectation conflicts finally surface when patients anticipate rapid symptom relief while providers pursue incremental, long-term disease control [63,200,206,220,245]. Postoperative pain management, chronic disease titration, and behavioral-change interventions are particularly susceptible to this tension: patients equate therapeutic success with immediate absence of discomfort, whereas clinicians focus on gradual stabilization or prevention of relapse [33]. In one study, oncologists found that patients had unrealistic expectations in terms of treatment outcomes [220], which resulted in a communication breakdown.

Longitudinal surveys show that uncorrected expectation gaps predict lower satisfaction scores, premature discontinuation of therapy, and a rise in “doctor-shopping” behaviors [207,249]. In maternity care, the belief that regular translation services are enough often conflicts with the actual needs of women. They expect a more collaborative and culturally connected way of communicating. This requires bicultural research assistants to fill the gap that standard interpreters cannot cover [83]. Conversational techniques that surface timelines explicitly, for example, “What improvements would you hope to see by next month?” significantly narrow these discrepancies and enhance adherence, particularly in multicultural settings where cultural time horizons vary [36,194].

#### Impacts of Communication Barriers

After identifying the various barriers that impede communication between patients and providers, it is important to understand the impacts of these barriers. This subsection addresses how these barriers affect patient care and health outcomes. A summary of the observed impacts across emotional, clinical, and systemic domains is presented in Table 5.

**Table 5.** Synthesis of the adverse clinical and systemic impacts of communication barriers. This table summarizes the compounding negative outcomes of linguistic, cultural, psychological, and cognitive barriers identified in the scoping review. Impacts are categorized by their effects on patient emotional experience, health care access, clinical decision-making (eg, misdiagnoses and delayed treatments), and systemic health efficiencies.

Domain affected	Key effects	References
Patient experience	Increased anxiety, distress, and emotional withdrawal due to unclear or poor communication.	[29,49,52,88,98,105,118,119,121-123,125-129,132,133,135-139,141,151-153,158,166,171,176,177,191-193,196-199,201-203,211-213,218,223,231-235,241,243,250-255]
Health care access	Delayed care-seeking, missed appointments, and reduced engagement due to stigma and language barriers.	[14,15,61,69,76,81-83,86,106-108,118-123,125-128,130,132-134,138,140-142,148,151,153,171,176,191-193,196-199,202,203,208,210,219,223,231-233,235,243,253,254,256-259]
Clinical decision-making	Misdiagnosis, delayed treatment, and poor shared decision-making from misunderstandings and mental model mismatches.	[51,63,81,93,99,104,118-120,122,124,125,127-129,132,133,135,137,138,151,152,161,191-193,196,197,202,203,206,210,212,213,217,219,223,232-235,241-243,254,255,260-263]
Health outcomes and system efficiency	Poorer health outcomes, increased readmissions, and diminished trust lead to systemic inefficiencies.	[62,64,87,89,91-93,98-101,118,120-122,124-126,128-130,132-134,138,140,141,146,151,153,171,191,193,196-198,201,203,210,211,217,223,231-235,241,243,253-255,264-271]

### What Are the Effects on Patient Experience and Health Care Outcomes?

Unclear or poorly structured communication can significantly impact patients, leading to increased anxiety and confusion and ultimately causing emotional withdrawal that affects future interactions. In oncology and acute-care contexts, studies have shown that ambiguous explanations of prognosis or treatment options are associated with a 20%‐30% increase in state anxiety scores compared to encounters that include structured signposting and clear, empathetic summaries [29,166]. Patients often internalize this uncertainty, creating “self-centered barriers.” They may prefer silence over seeking clarification. For example, patients with breast cancer often say that their own beliefs and feelings of embarrassment make it harder for them to talk about sexual health issues than any specific actions by their health care providers [177]. Likewise, women with hearing loss might go to great lengths to hide their impairment to avoid social stigma. This can lead to pretending they understand instead of truly engaging [88]. Patients exposed to such communication patterns often choose silence over clarification, fearing that questions may be perceived as inappropriate or burdensome [52,105].

These challenges are further exacerbated for LEP patients, who struggle not only with direct language translation but also with comprehending idiomatic expressions, medical jargon, and cultural references embedded in clinician speech. As documented by researchers [148,158,218,251], LEP patients frequently report feelings of shame, low self-confidence, and guilt about demanding resources, such as interpreters. This emotional burden contributes to a pattern of passivity during consultations, in which patients listen but do not actively engage unless explicitly prompted [158,251]. This passivity often comes from a deep feeling of “powerlessness.” Non–English-speaking patients in acute care say their struggle to navigate the dominant language and cultural norms makes them vulnerable to racism and exclusion. This reinforces their silence [69]. For immigrant groups such as Brazilians, this fear goes beyond the clinic walls. Their undocumented status and strong mistrust of authority figures result in a total avoidance of formal health care options [98]. Moreover, the psychological impact of feeling overlooked or excluded can erode trust in health care providers and foster long-term dissatisfaction with the care system. Language barriers, especially when left unaddressed, are strongly linked to treatment discontinuation, missed follow-ups, and medication errors stemming from misinterpreted instructions [252]. These compounding effects initiate a detrimental feedback loop: poor communication elevates emotional vulnerability, reducing the likelihood of future engagement and increasing the risk of adverse health outcomes.

Communication barriers also manifest as concrete impediments to health care outcomes. Language differences, persistent stigma, and fear of social judgment often discourage individuals, especially migrants and members of socially marginalized groups, from seeking timely medical attention [14,15]. These barriers not only reduce appointment adherence but also contribute to significant delays in help-seeking behaviors, leaving many at greater risk for untreated health conditions [256]. Growing evidence from mixed-methods research highlights the profound impact of perceived clinician-patient distance. Patients who feel that their health care providers are culturally or linguistically unapproachable are twice as likely to miss follow-up appointments and are significantly less inclined to use preventive health care services [256,257]. Qualitative interviews in community clinics reveal that the internalized shame linked to LEP deters patients from scheduling nonurgent consultations until conditions become acute [14,15].

At the point of care, communication breakdowns extend their influence to the core of clinical decision-making. Different mental models can lead to misunderstandings about disease origins or treatment expectations, as well as confusing diagnostic reasoning. This misalignment frequently leads to misclassifying symptoms and unnecessary delays in initiating therapy [146,260,269]. For instance, people who use drugs often postpone seeking care for skin and soft tissue infections because of anticipated judgment, whereas having a trusted, nonjudgmental physician is associated with lower infection rates [217]. Similarly, African American and Hispanic women with a history of gestational diabetes reported overwhelming communication and insufficient guidance for type 2 diabetes prevention, limiting behavior change despite the patients’ interest [219]. In chronic disease management, spiritual illness beliefs and distrust of biomedical protocols can further reduce disclosure, adherence, and shared decision-making [261,262].

The far-reaching consequences of these communication gaps are reflected in measurable declines in individual health outcomes and overall system efficiency. Cohort studies consistently link ineffective clinician-patient communication to higher 30-day readmission rates and lower disease-specific quality-of-life scores, even when controlling for comorbidities and socioeconomic factors [264,268]. For birthing people of color in the United States, poor communication and neglect are reported as key factors behind birth inequities, especially in the postpartum period. This shows how communication problems can harm patient safety and experience [87]. Similarly, for migrant women in Portugal, having limited language skills in the host country is linked to a lower perceived quality of prenatal care. This indicates that without help with language, the system cannot provide fair perinatal health outcomes [89].

Poor communication in health care leads to higher use of resources. This results in duplicate tests, unnecessary emergency visits, and longer hospital stays. These issues add extra pressure to already tight health care budgets [265,266]. Importantly, repeated negative interactions between patients and providers can erode public trust in health care systems, undermining the foundation for the successful uptake of preventive services and adherence to chronic care regimens [267].

### How Does Each Barrier Impact Patient Care and Health Outcomes?

#### Overview

Linguistic barriers, such as LEP and lack of access to professional interpreters, directly undermine patient comprehension. Patients may misinterpret diagnoses, treatment instructions, or follow-up plans, leading to emotional withdrawal and heightened anxiety [29,103,166]. This isolation is especially strong for older immigrants. Research shows that older adult patients with language barriers often experience a “double burden” of being cutoff from both language and digital resources. This situation makes it hard for them to access telehealth services or understand medication management without depending heavily on family members [80]. Language discordance has also been linked to delayed care-seeking and missed appointments, as patients feel ill-equipped to articulate their needs [14,15,61]. Moreover, language difficulties can hinder accurate diagnosis and shared decision-making, contributing to clinical errors [62,272,273]. Cultural barriers exacerbate these issues by creating mismatches in expectations and values. For example, culturally rooted discomfort in discussing illness with providers of a different gender or preference for family-centered decision-making may inhibit transparency during consultations [86,176]. These dynamics delay engagement with care, reduce adherence, and generate systemic inefficiencies [51,62,256].

Psychological barriers, including stigma, anxiety, and emotional overload, particularly affect patients dealing with chronic or mental health conditions. These patients may withhold critical information, leading to misdiagnosis and treatment delays. Research on HIV prevention indicates that fear of social rejection stops at-risk groups from obtaining the treatments they need, indicating the need for both clinical and focused efforts on building trust in the community to be effective [93]. Emotional distress also impairs memory and verbal communication during clinical encounters, diminishing both patient experience and diagnostic clarity [220]. MMDs further complicate communication. These mismatches undermine trust, disrupt shared decision-making, and increase patient dissatisfaction. Patients may misinterpret medical reasoning as dismissive or unresponsive, leading to poor adherence and repeated consultations [33,206,262]. A clear example of this is seen in chronic pain management. Patients often want immediate relief, following the “cure” model. In contrast, clinicians concentrate on functional improvement, using the “management” model. This unrecognized difference causes mutual frustration, prompting “doctor shopping” and damaging the therapeutic relationship [87].

#### Interventions to Address Barriers

After identifying the key communication barriers and their consequences, we now turn to the interventions developed to mitigate them. This section examines the intervention strategies that have been implemented to address communication barriers and identifies which barrier types they primarily target. Drawing from the literature, we categorize these interventions and their targeted barriers and depict them in Table 6.

**Table 6.** Overview of intervention strategies targeting specific health care communication barriers. This table shows human-centered and technological interventions, such as professional interpreters, AI-powered multilingual chatbots, empathy training, and wearable monitors, along with the specific patient-provider barriers they address. It assesses the reported effectiveness of these solutions based on the reviewed empirical literature.

Intervention type	Barriers targeted	Effectiveness summary	References
Professional interpreters	Language barriers	Reduces misdiagnosis and increases satisfaction among LEP patients.	[14,15,51,73,76,77,83,90,96,118-123,125-130,132,134-139,141,142,145,146,150,153,161,272,274-276]
AI-powered chatbots	Language barriers	Increases triage efficiency and multilingual engagement. Still evolving in clinical accuracy.	[57,100-102,243,277,278]
Multilingual translation tools (AI-based)	Language barriers	Bridges language gaps; effectiveness depends on translation accuracy and context awareness.	[79,100,110,118-120,175,235,279-281]
Cultural competency training	Cultural barriers	Enhances trust and adherence in diverse populations.	[50,69,118,122,123,125,127,132,133,135,141,175,183,191,192,196,199,202,235,282-284]
Empathy training	Psychological barriers	Reduces stigma; improves mental health engagement.	[70,118,122,125,132,133,135,151,191,196,202,203,215,285-289]
Adopting plain language and visual aids	Mental model differences	Improves patient comprehension, reduces miscommunication, and enhances adherence.	[55,62,70,76,118-122,125,129,131-133,136-138,141,144,149,153,171,191,195-198,212,213,232,242,243,251,253,255,272,273,290,291]
Wearable devices and remote monitoring	All barriers	Enables continuous nonverbal care and real-time data transmission.	[80,81,91,193,231,235,243,253-255,258,270,271,292,293]

Professional language‐service interventions remain the most consistently validated strategy for mitigating communication breakdowns with LEP populations. Evidence shows that trained interpreters reduce diagnostic errors, improve satisfaction, lower readmissions, and support better maternal and patient outcomes compared with ad hoc or family interpreters [14,15,146,150]. Bicultural research assistants may further improve engagement when language and cultural needs intersect [83]. Decision-analysis models further indicate that interpreter use yields cost savings through shorter lengths of stay and fewer duplicate tests, largely because accurate histories are obtained on first contact [272]. Complementary AI-based multilingual translation tools extend this reach to low-resource or time-critical settings, with early trials demonstrating rapid phrase-level accuracy above 90% for high-volume languages, though performance drops in idiomatic or domain-specific contexts [280]. The emerging consensus is that hybrid models, professional interpreters for complex consultations, and vetted machine translation for routine exchanges, optimize both cost and clinical fidelity [49-52,161,175,281].

Emerging health care AI shows the shift from basic translation tools to “AI notetaking” agents that can translate and organize clinical notes in real-time [110,175]. These tools aim to lessen the administrative stress that often causes rushed and low-quality communication in multilingual environments. Additionally, hybrid methods that combine large language models with traditional Bayesian networks are now more accurate than human psychologists in certain vignette-based diagnostic tasks. This suggests a future where AI serves as a backup to help avoid communication-related diagnostic mistakes. AI-powered chatbots offer a scalable front line for triage and information dissemination, particularly in multilingual or resource-limited environments. Importantly, chatbot interfaces equipped with real-time translation and culturally adaptive scripts demonstrate higher engagement rates among LEP users than static FAQ (frequently asked question) pages [277]. Nevertheless, concerns persist regarding diagnostic accuracy, bias propagation, and data privacy, underscoring the need for rigorous validation against gold-standard clinical protocols [57,277]. Ongoing research and development is focusing on integrating fallback loops with human clinicians and incorporating evidence-based symptom-checker algorithms to enhance reliability [278].

Cultural barriers often arise from mismatched values, health beliefs, or discomfort tied to identity differences. These challenges are best addressed through cultural competency training that combines classroom instruction with hands-on experiences. Cultural competency interventions improve providers’ ability to align care with patients’ sociocultural contexts, thereby enhancing trust, communication quality, and care equity [50,175,183]. Programs featuring community immersion and scenario-based simulations have been shown to improve medication adherence and follow-up rates in ethnically diverse groups. These outcomes are largely driven by increases in perceived clinician trustworthiness [282]. For example, a mixed-methods study of Indian physiotherapy students found that a targeted cultural competency training program significantly improved therapists’ ability to engage in patient-centered care, leading to a measurable increase in perceived treatment adherence [294]. Participatory and system-level approaches further demonstrate that culturally responsive service design can address structural barriers and improve engagement among underserved populations [53]. Recent cluster trials applying intersectional frameworks, which account for overlapping identities such as race, gender, and socioeconomic status, have further enhanced shared decision-making outcomes, especially in chronic disease management [283]. Qualitative research backs this approach by showcasing the unique needs of specific groups. For example, African American, Hispanic, and Appalachian women with a history of gestational diabetes all said that communication with providers often felt “overwhelming” and did not provide practical advice. This suggests that interventions should be designed to address not only cultural backgrounds but also the specific socioeconomic challenges, such as cost and transportation, that these groups face together [219]. Notably, evidence suggests that sustained, context-specific training yields more durable improvements than one-time workshops, highlighting the need for long-term institutional investment in cultural responsiveness.

Interventions that target provider attitudes and emotional responsiveness, particularly empathy training, are central to addressing psychological barriers such as stigma, fear, and emotional reluctance. Structured empathy training can reduce stigmatizing language, increase the likelihood of mental health referral uptake among marginalized patients, and strengthen reflective listening [70,215,285-289]. By fostering a safer emotional environment, such training improves disclosure of sensitive issues, particularly in stigmatized contexts. For people who inject drugs, having a “trusted doctor,” defined by nonjudgmental communication and emotional safety, was the strongest predictor for seeking timely care for soft tissue infections [217].

MMDs often arise from misaligned expectations, reasoning styles, or divergent understandings of illness between patients and health care providers. To bridge these gaps, clinicians should use clear, direct language and avoid medical jargon, acronyms, or idiomatic expressions. Using short sentences with frequent pauses can facilitate information processing, particularly in interpreted encounters [144]. Verbal explanations should be supplemented with visual aids, such as diagrams, illustrated instructions, or step-by-step graphics, to enhance patient comprehension [70,144,251]. However, even these tools must be designed with the user’s mental model in mind. A notable example comes from maternal health programs in Nigeria [229]. Researchers found that simply copying Western information formats did not work for women with low or no literacy. Instead, successful programs needed a community-based design process. This approach treated these women not just as patients, but as co-designers who could shape the information to fit their communal ways of understanding [229]. These strategies are particularly beneficial for individuals with limited health literacy or limited familiarity with biomedical concepts [144,195,291]. Evidence from decision-aid trials in primary care indicates that aligning patient and provider mental models through visual communication and iterative teach-back methods significantly reduces diagnostic errors and increases concordance in treatment planning [272,273]. In maternity care, this connection works best through “reflexive collaboration.” Bicultural research assistants help close the gap between clinical protocols and the real experiences of women with limited English skills. This ensures that the “care partnership” is more than just a policy ideal; it becomes a practical reality [83].

Finally, wearable devices and remote-monitoring platforms present a complementary avenue for circumventing verbal, cultural, psychological, and cognitive barriers by privileging objective, continuous physiologic data. Studies in cardiac and diabetic populations reveal that real-time telemetry, transmitted directly to care teams, reduces unscheduled hospital visits by up to 25% and facilitates earlier intervention independent of patients’ communicative proficiency [293]. The latest generation of wearables integrates haptic alerts and multilingual smartphone dashboards, allowing patients to interpret feedback in their preferred language while clinicians receive standardized data streams [292]. This will significantly help reduce communication barriers as patient data will be readily available to doctors. As a result, the context of the patient’s illness can be more easily understood, especially with the help of AI models. Thus, as interoperability standards and AI capabilities develop, these technologies are set to become key parts of overall plans that tackle all aspects of communication barriers.

## Discussion

### Overview

This scoping review aimed to map the existing literature to identify key communication barriers in patient-provider settings, evaluate their impacts on clinical outcomes, and explore targeted intervention strategies. Relative to these objectives, our synthesis reveals that clinical communication is predominantly disrupted by four interconnected barrier types: linguistic, cultural, psychological, and MMDs. The literature indicates that these barriers rarely occur in isolation; rather, they interact and compound to precipitate adverse clinical events, ranging from delayed care-seeking to diagnostic errors and emotional distress. Furthermore, while various human-centered and technological interventions have been deployed to mitigate these challenges, our findings highlight a significant gap in the literature regarding real-time, adaptive communication support.

### Summary of Evidence

#### Principal Findings

The review shows a steady rise in publications about communication barriers in health care, which increased markedly from 2021. This surge in research activity coincides with the onset of the COVID-19 pandemic, which intensified global scrutiny of systemic weaknesses in health care, including the critical role of communication barriers [161,295]. The trend reflects a growing recognition of communication as a key determinant of health care quality and an area ripe for interdisciplinary investigation [60].

This review breaks down communication barriers into four main types: language, cultural, psychological, and MMDs. Each type highlights specific but overlapping causes of problems in patient-provider interactions. While the focus of the literature has largely been on language and cultural barriers, there has been a growing body of research on how MMDs and psychological barriers may manifest as communication barriers in health care [49,50]. The distribution highlights an opportunity for more balanced, multifaceted research agendas that move beyond language proficiency to capture the full spectrum of communicative impediments in clinical care.

Interestingly, while distinct communication barriers exist, they do not seem to be linked to specific clinical or emotional outcomes. Instead, multiple barriers often happen together and contribute to negative experiences. For example, studies have found that patients dealing with both language and psychological challenges often report poor interactions with their health care providers [51,158,218,251]. These overlapping barriers can also delay the decision to seek care, as patients may avoid medical visits due to fear, stigma, or difficulty in communicating effectively [52,60]. Notably, patients in acute care settings frequently describe a profound sense of “powerlessness” when linguistic barriers combine with cultural oversight [69,183]. In the context of managing chronic diseases, differences in mental models and cultural beliefs make care more complicated. Patients who believe their illness has spiritual causes may dismiss biomedical explanations, leading to low adherence and disengagement [260-262]. Therefore, communication barriers, regardless of their type, often lead to clinical and emotional effects. This highlights the need to address them in a unified and thorough way. Our analysis in this provides a high-level overview of reported impacts; however, future reviews should distinguish among patient-, clinician-, and system-level outcome domains to build a more nuanced understanding [61]. Such stratification would require different data extraction procedures and an explicit logic model.

A wide range of interventions was identified, including traditional solutions such as professional interpreter services and cultural competency training, as well as newer developments in AI-assisted communication. Professional interpreters have been shown to reduce diagnostic errors and improve satisfaction among LEP patients [14,15,103,272]. However, in the absence of formal services, reliance on multilingual staff is often viewed internally as a “gift” to colleagues and families rather than a systemic solution, highlighting a gap in sustainable support [51,62]. Additionally, empathy and stigma-reduction training improve mental health engagement [63,288,289]. The emergence of AI has also created innovations with the rise of AI-powered chatbots and multilingual tools that have shown promise for triaging and supporting routine patient interactions [208,235]. However, many of these tools still lack contextual awareness or real-time adaptability. This limits their effectiveness in complex encounters [206,277]. The overall results of this review highlight the need to study the interplay between various barriers to better understand the impacts and to properly mitigate those aforementioned impacts.

#### Interaction Between Barriers, Interventions, and Effects

Communication barriers in health care rarely operate in isolation. Instead, linguistic, cultural, psychological, and cognitive barriers often interact in complex ways, compounding their effects on patient outcomes [161,175]. For example, a language barrier may initially prevent accurate expression of symptoms, but this can be exacerbated when the patient also experiences psychological discomfort, such as anxiety, stigma, or fear of judgment [69]. This compounding effect is clear in critical care settings. During the COVID-19 pandemic, physical communication challenges increased psychological distress. This created a cycle of frustration and fear that harmed recovery. This leads to further reluctance to engage with providers [15,32]. Even with interpreter services, MMDs may persist when patients conceptualize illness or treatment through culturally grounded frameworks that diverge from biomedical reasoning [33,52,260]. Studies show that this often leads to a deep feeling of “powerlessness.” Patients feel unable to handle both the language barrier and the cultural hierarchy of the hospital environment [69]. This interplay of barriers contributes to misdiagnosis, reduced treatment adherence, emotional distress, and, ultimately, a breakdown of trust in the patient-provider relationship [166].

Interventions must be designed with this interconnectedness in mind. While professional interpreters are effective in addressing language gaps, their impact improves significantly when coupled with cultural competency training that enables providers to recognize patients’ sociocultural beliefs and adapt communication accordingly [61,282]. Empathy training also plays a critical role by addressing both psychological safety and cognitive attunement, helping providers to better navigate emotional sensitivities and differing expectations [103,166,220]. In parallel, the use of plain language and visual aids has shown promise in bridging both linguistic and cognitive gaps, especially among patients with limited health literacy or those unfamiliar with medical reasoning [70,144,251]. For example, using low-tech strategies such as simple communication boards and video interpretation was crucial for overcoming various barriers during the pandemic [75].

Ultimately, the effectiveness of any single intervention is contingent on its ecological fit. That is to say, how well it aligns with and addresses the interdependent nature of barriers present in each clinical encounter. Integrated, multilevel strategies, which combine human-centered training, language access tools, and communication aids, are more likely to produce sustained improvements in trust, satisfaction, and clinical outcomes.

#### The Mechanisms and Outcomes of Communication Barriers

We map together repeated evidence into a transparent chain that explains how communication failures spread from minor interaction breakdowns to clinical and experiential harms. Multimedia Appendix 3 [33,36,46-48,51,58-60,64-67,69-72,84-87,91,105,106,108,135-137,143,144,154-160,164-169,172-174,176,178-182,186-188,194,200,207,214,215,217,224-227,236-239,244,249] explains it in more detail.

First, language barriers often lead to a loss of accurate information. For patients with LEP who lack interpreter support, this can lead to failures to express symptoms or to understand clinical instructions. This results in misdiagnoses, medical errors, and missed warning signs, as shown in studies on language mismatch and LEP [46-48,58,59,65-67,70-72,104,143,144]. When communication depends on ad hoc interpreters, the issue shifts from no translation to filtering. This filtering, often carried out by family members without medical vocabulary, can result in key symptoms being omitted and violations of confidentiality [64,154-160,164-169]. Together, these issues illustrate why language differences are often linked to serious safety problems. The clinical record is based on incomplete, distorted, or socially filtered information. Second, cultural barriers mainly affect interactions by creating mismatches in meaning and undermining relationships. Belief-system conflict directly leads to conflicts between cultural or religious norms and biomedical guidance. This conflict is linked to nonadherence and resistance to care [106,144,172-174,188,200]. Nonverbal misinterpretation is the second mechanism. Different interpretations of tone, gesture, or other signals can create feelings of disrespect, which heightens resistance to care [85,91,143,178,179,194]. Lastly, conflicts over authority and disclosure norms lead to a sense of disrespect and disregard for family hierarchy. This can result in feelings of powerlessness and perceptions of racism [69,86,87,180-182,186,187]. This pathway clarifies that cultural barriers go beyond simple preference differences. They can disrupt the social conditions needed for effective communication and acceptance of recommendations.

Third, psychological barriers work by hindering disclosure and disrupting narrative clarity when individuals feel emotionally threatened. Stigma is clearly linked to fear of judgment and to hiding relevant history, leading to the withholding of sensitive information [60,84,176,187,214,215,217]. Emotional overload and discomfort, such as distress, fear, anxiety, and low confidence, relate to fragmented or incomplete information. This can directly lower the quality of clinical judgment and triage [51,108,224-226]. Psychological safety also serves as a factor that anticipates dismissal or intimidation, leading to withdrawal from interaction [86,143,186,227-229]. Overall, these findings highlight a mechanism-focused view: emotional and social threats limit what patients can or will share, thereby affecting the content and completeness of the clinical information available for decision-making. Finally, MMDs lead to adverse outcomes through mismatched reasoning, assumptions, and expectations about time. A reasoning gap is the difference between how the general public understands a situation and how clinical diagnostics work [143,227,236-239]. This gap can cause decision-making paralysis and communication issues. Assumption divergence, or differing preconceived ideas about illness and responsibilities, is linked to patients feeling that their provider does not care [168,237-239,244]. Expectation gaps, especially conflicts between patients seeking a cure and clinicians focused on management, lead to frustration and reinforce the perception that providers lack compassion [33,36,194,207,249]. This explains why dissatisfaction and disengagement can continue even when people seem to understand what is being said. Thus, the main issue is not the words themselves, but the alignment between explanations and goals.

In summary, the reviewed evidence is combined into a connected mechanism-outcome framework. It shows that communication barriers are not just isolated inconveniences. They are systematic disruptions that influence what information is shared, how it is understood, and whether clinical relationships can support shared decision-making.

#### Policy Implications

The findings of this review have several important implications for policy that extend beyond individual clinical encounters. They highlight the need for broader changes in health care systems. First, the strong and ongoing impact of language barriers shows we need language-access policies to ensure professional interpreters are available [51,60,69,109]. These policies should also regulate the use of AI translation tools to maintain accuracy and safety [57,100-102]. Second, the identified cultural and psychological barriers point to the importance of developing the workforce. Policies should include long-term programs in cultural humility, empathy training, and communication skills as part of clinician licensing and ongoing education requirements [50,69,135,202]. Third, the evidence about different mental models indicates a need for policies that encourage the creation and use of plain-language materials, visual aids for decision-making, and communication standards that are sensitive to health literacy in all care settings [55,62,70,76,122]. Finally, with the rise of AI, policymakers should back regulations that encourage responsible innovation and tackle equity issues [57,277,278]. They should demand more transparency in the model training and development process. AI models should be regularly checked for bias, and human oversight should be ensured. Together, these policy directions can lead to more fair, safe, and effective communication practices, helping to reduce the systemic disparities noted in this review.

### Limitations

This scoping review has several methodological limitations that should be taken into account. First, because of the nature of a scoping review, our main goal was to outline the range of literature instead of assessing how effective specific interventions are. As a result, this review cannot make clear claims about the clinical success rates of the identified technological or human-centered solutions. Second, our findings are constrained by the mostly retrospective nature of the evidence. Most of the studies we included relied on postencounter surveys, qualitative interviews, or recorded consultations. This limits our ability to capture real-time physiological and nonverbal signals, such as changes in heart rate variability or facial expressions, related to communication breakdowns as they happen.

### Conclusions

There is increasing focus in both research and clinical practice on the many layers of communication barriers in health care. These challenges extend beyond simple language issues. As health care systems grow more diverse and technology-focused, grasping the intricate connections between language, psychological, cognitive, and cultural barriers is becoming more important. This change shows a deeper understanding of how these barriers work together to threaten patient safety, affect clinical decisions, and limit fair access to care, particularly for marginalized and vulnerable groups. Unlike existing literature reviews, which typically examine communication barriers separately and rely on past data, this scoping review takes a new approach. It sets itself apart by creating a unified framework that connects the combined effects of language, culture, psychology, and thinking barriers directly to negative clinical outcomes. By introducing this pathway, the review fills an important gap between traditional patient-focused interventions and new digital health technologies. In the real world, these findings have significant implications. This framework acts as a guide for designing and implementing adaptive, AI-supported communication systems and real-time monitoring tools. By combining these advanced tools with empathetic clinical interactions and supportive policies, health care systems can better identify patient distress, reduce avoidable medical errors, and address systemic inequalities. Ultimately, making health care communication responsive and patient-focused is essential for providing fair, high-quality care in a more diverse, digitally connected clinical environment.

## Supplementary material

10.2196/79744 Multimedia Appendix 1 Operational definitions of key communication concepts and the four primary barrier categories (linguistic, cultural, psychological, and mental models) used in the systematic review.

10.2196/79744 Multimedia Appendix 2 Database-specific search histories, including exact search strings and applied filters copied from PubMed, IEEE Xplore, CINAHL, and Current Contents Connect, for searches conducted from January 1, 2004, to April 30, 2026.

10.2196/79744 Multimedia Appendix 3 Detailed matrix mapping specific communication barriers to their mechanisms of action and the resulting adversarial clinical outcomes.

10.2196/79744 Checklist 1 PRISMA-ScR Checklist.

## Abbreviations

FAQ: frequently asked question
MMD: mental model difference
PRISMA-S: Preferred Reporting Items for Systematic Reviews and Meta-Analyses literature search extension
PRISMA-ScR: Preferred Reporting Items for Systematic Reviews and Meta-Analyses extension for Scoping Reviews
