# Supplementary material for: Communication Barriers in Patient-Provider Interactions in Health Care: Scoping Review
Source: J Med Internet Res. 2026 Jul 21;28:e79744. doi: 10.2196/79744 (PMC13387742; doi:10.2196/79744)
Supplement: Multimedia Appendix 1 — Operational definitions of key communication concepts and the four primary barrier categories (linguistic, cultural, psychological, and mental models) used in the systematic review. [file jmir-v28-e79744-s001.docx]

**Table S1.** Operational definitions of key communication concepts and the four primary barrier categories (Linguistic, Cultural, Psychological, Mental Models) utilized in the systematic review.

| **Term** | **Definition** |
| --- | --- |
| Communication | Communication is the process where people share information, thoughts, and feelings through verbal, non-verbal, and symbolic methods, all within a social and cultural setting. |
| Effective Communication | Effective communication can be described as when a communication is understood and acted upon as intended. |
| Communication Failure | Communication failure occurs when effective communication cannot occur due to some impediments. |
| Communication Barriers | Obstacles that hinder effective communication. |
| Language Barriers | Impediments that emerge whenever patients and clinicians lack a shared linguistic repertoire or a common foundation of health literacy. |
| Cultural Barriers | Obstacles rooted in divergent belief systems, values, social norms, and religious prescriptions that shape how illness is conceptualized, how authority is negotiated, and which therapeutic options are deemed acceptable. |
| Psychological Barriers | Barriers arising from intrapsychic states that limit a patient’s willingness or ability to disclose sensitive information or participate in shared decision-making. |
| Mental Model Difference | Cognitive incongruities between patients and healthcare professionals that stem from disparate educational backgrounds, experiential histories, and cultural frames of reference. |
