# Supplementary material for: Communication Barriers in Patient-Provider Interactions in Health Care: Scoping Review
Source: J Med Internet Res. 2026 Jul 21;28:e79744. doi: 10.2196/79744 (PMC13387742; doi:10.2196/79744)
Supplement: Multimedia Appendix 2 — Database-specific search histories, including exact search strings and applied filters copied from PubMed, IEEE Xplore, CINAHL, and Current Contents Connect, for searches conducted from January 1, 2004, to April 30, 2026. [file jmir-v28-e79744-s002.docx]

**Table S1.** Search terms and exact strings utilized to retrieve empirical literature on patient-provider communication barriers from PubMed. The search was conducted from Jan 1 2004 to Apr 30 2026.

| **Scope** | **Search Terms** |
| --- | --- |
| General Search | ("communication barriers") AND ("patient-provider") NOT ("review" OR "systematic review" OR "meta-analysis") *Filters: English, from 2004/1/1 - 2026/4/30 Sort by: Most Recent* |
| General Search | (("language barriers" OR "cultural barriers" OR "psychological barriers" OR "technological barriers")  AND ("healthcare" OR "clinical communication" OR "hospital interactions"))  AND ("patient outcomes" OR "diagnostic accuracy" OR "treatment adherence")  NOT ("review" OR "systematic review" OR "meta-analysis") *Filters: English, from 2004/1/1 - 2026/4/30 Sort by: Most Recent* |
| Language Barriers | ("language barriers" OR "limited English proficiency" OR "non-native speakers" OR "translation issues" OR "interpreter services") AND (("Delivery of Health Care"[MeSH]) OR "healthcare" OR "medical settings" OR "clinical communication") AND (("Treatment Outcome"[MeSH] OR "Patient Satisfaction"[MeSH]) OR ("patient outcomes" OR "satisfaction")) NOT ("review" OR "systematic review" OR "meta-analysis") *Filters: English, from 2004/1/1 - 2026/4/30 Sort by: Most Recent* |
| Cultural Barriers | (("Cultural Competency"[MeSH] OR "Health Disparities") OR ("cultural barriers" OR "cross-cultural communication" OR "ethnic disparities" OR "belief systems")) AND (("Delivery of Health Care"[MeSH]) OR "healthcare" OR "hospital communication" OR "clinical interactions") AND (("Medication Adherence"[MeSH]) OR "treatment adherence" OR "patient trust") NOT ("review" OR "systematic review" OR "meta-analysis") *Filters: English, from 2004/1/1 - 2026/4/30 Sort by: Most Recent* |
| Psychological Barriers | (("Stress, Psychological"[MeSH] OR "Anxiety"[MeSH]) OR "psychological barriers" OR "emotional challenges" OR "fear of disclosure" OR "stress" OR "stigma") AND ("Professional-Patient Relations"[MeSH] OR "patient-provider communication") AND ("Treatment Outcome"[MeSH]) NOT ("review" OR "systematic review" OR "meta-analysis") *Filters: English, from 2004/1/1 - 2026/4/30 Sort by: Most Recent* |
| Technological Intervention | (("Telemedicine"[MeSH] OR "Health Information Technology") OR ("technological barriers" OR "digital divide" OR "e-health communication")) AND (("Delivery of Health Care"[MeSH]) OR "healthcare delivery" OR "remote consultations") AND (("Patient Participation"[MeSH]) OR "patient engagement") NOT ("review" OR "systematic review" OR "meta-analysis") *Filters: English, from 2004/1/1 - 2026/4/30 Sort by: Most Recent* |
| Impacts of Communication Barrier | ("communication barriers" OR "Communication"[MeSH] OR "Language Barrier" OR "Cultural Competency"[MeSH]) AND ("Treatment Outcome"[MeSH] "health outcomes" OR "patient satisfaction" OR "treatment adherence" OR "emotional well-being") AND ("Health Services"[MeSH] OR "healthcare" OR "clinical settings") NOT ("review" OR "systematic review" OR "meta-analysis") *Filters: English, from 2004/1/1 - 2026/4/30 Sort by: Most Recent* |
| Interventions | ("Intervention Studies" OR "Patient-Centered Care"[MeSH])  AND ("Language Barrier" OR "Cultural Competency"[MeSH] OR "Stress, Psychological"[MeSH] OR "Telemedicine"[MeSH])  AND ("Delivery of Health Care"[MeSH] OR "healthcare" OR "hospital communication" OR "clinical interactions")  NOT ("review" OR "systematic review" OR "meta-analysis") *Filters: English, from 2004/1/1 - 2026/4/30 Sort by: Most Recent* |
| Mental Model Differences | ("Models, Psychological"[MeSH] OR "mental models" OR "cognitive models" OR "perceptual differences") AND ("healthcare" OR "clinical settings" OR "patient-provider communication" OR "Professional-Patient Relations"[MeSH]) NOT ("review" OR "systematic review" OR "meta-analysis") *Filters: English, from 2004/1/1 - 2026/4/30 Sort by: Most Recent* |

**Table S2.** Search terms and exact strings utilized to retrieve empirical literature on patient-provider communication barriers from IEEE Xplore. The search was conducted from Jan 1 2004 to Apr 30 2026. English Journal articles were filtered in the screening process. Date filters were applied manually.

| **Scope** | **Search Terms** |
| --- | --- |
| General Search | ("communication barriers") AND ("patient-provider") NOT ("review" OR "systematic review" OR "meta-analysis") |
| General Search | (("language barriers" OR "cultural barriers" OR "psychological barriers" OR "technological barriers")  AND ("healthcare" OR "clinical communication" OR "hospital interactions"))  AND ("patient outcomes" OR "diagnostic accuracy" OR "treatment adherence")  NOT ("review" OR "systematic review" OR "meta-analysis") |
| Language Barriers | ("language barriers" OR "limited English proficiency" OR "non-native speakers" OR "translation issues" OR "interpreter services") AND (("Delivery of Health Care") OR "healthcare" OR "medical settings" OR "clinical communication") AND (("Treatment Outcome" OR "Patient Satisfaction") OR ("patient outcomes" OR "satisfaction")) NOT ("review" OR "systematic review" OR "meta-analysis") |
| Cultural Barriers | (("Cultural Competency" OR "Health Disparities") OR ("cultural barriers" OR "cross-cultural communication" OR "ethnic disparities" OR "belief systems")) AND (("Delivery of Health Care") OR "healthcare" OR "hospital communication" OR "clinical interactions") AND (("Medication Adherence") OR "treatment adherence" OR "patient trust") NOT ("review" OR "systematic review" OR "meta-analysis") |
| Psychological Barriers | (("Stress, Psychological" OR "Anxiety") OR "psychological barriers")  AND ("Professional-Patient Relations" OR "patient-provider communication")  AND ("Treatment Outcome")  NOT ("review" OR "systematic review" OR "meta-analysis") |
| Technological Intervention | (("Telemedicine" OR "Health Information Technology") OR ("technological barriers" OR "digital divide")) AND (("Delivery of Health Care") OR "healthcare delivery") AND (("Patient Participation") OR "patient engagement") NOT ("review" OR "systematic review" OR "meta-analysis") |
| Impacts of Communication Barrier | ("Communication" OR "Language Barrier" OR "Cultural Competency") AND "Treatment Outcome" AND "Health Services" NOT ("review" OR "systematic review" OR "meta-analysis") |
| Interventions | ("Intervention Studies" OR "Patient-Centered Care")  AND ("Language Barrier" OR "Cultural Competency" OR "Stress, Psychological" OR "Telemedicine")  AND "Delivery of Health Care"  NOT ("review" OR "systematic review" OR "meta-analysis") |
| Mental Model Differences | ("Models, Psychological" OR "mental models") AND "Professional-Patient Relations" NOT ("review" OR "systematic review" OR "meta-analysis") |

**Table S3.** Search terms and exact strings utilized to retrieve empirical literature on patient-provider communication barriers from CINAHL. The search was conducted from Jan 1 2004 to Apr 30 2026. English Journal articles were filtered in the screening process. Date filters were applied manually.

| **Scope** | **Search Terms** |
| --- | --- |
| General Search | ("communication barriers") AND ("patient-provider") NOT ("review" OR "systematic review" OR "meta-analysis") |
| General Search | (("language barriers" OR "cultural barriers" OR "psychological barriers" OR "technological barriers")  AND ("healthcare" OR "clinical communication" OR "hospital interactions"))  AND ("patient outcomes" OR "diagnostic accuracy" OR "treatment adherence")  NOT ("review" OR "systematic review" OR "meta-analysis") |
| Language Barriers | ("language barriers" OR "limited English proficiency" OR "non-native speakers" OR "translation issues" OR "interpreter services") AND (("Delivery of Health Care") OR "healthcare" OR "medical settings" OR "clinical communication") AND (("Treatment Outcome" OR "Patient Satisfaction") OR ("patient outcomes" OR "satisfaction")) NOT ("review" OR "systematic review" OR "meta-analysis") |
| Cultural Barriers | (("Cultural Competency" OR "Health Disparities") OR ("cultural barriers" OR "cross-cultural communication" OR "ethnic disparities" OR "belief systems")) AND (("Delivery of Health Care") OR "healthcare" OR "hospital communication" OR "clinical interactions") AND (("Medication Adherence") OR "treatment adherence" OR "patient trust") NOT ("review" OR "systematic review" OR "meta-analysis") |
| Psychological Barriers | (("Stress, Psychological" OR "Anxiety") OR "psychological barriers")  AND ("Professional-Patient Relations" OR "patient-provider communication")  AND ("Treatment Outcome")  NOT ("review" OR "systematic review" OR "meta-analysis") |
| Technological Intervention | (("Telemedicine" OR "Health Information Technology") OR ("technological barriers" OR "digital divide")) AND (("Delivery of Health Care") OR "healthcare delivery") AND (("Patient Participation") OR "patient engagement") NOT ("review" OR "systematic review" OR "meta-analysis") |
| Impacts of Communication Barrier | ("Communication" OR "Language Barrier" OR "Cultural Competency") AND "Treatment Outcome" AND "Health Services" NOT ("review" OR "systematic review" OR "meta-analysis") |
| Interventions | ("Intervention Studies" OR "Patient-Centered Care")  AND ("Language Barrier" OR "Cultural Competency" OR "Stress, Psychological" OR "Telemedicine")  AND "Delivery of Health Care"  NOT ("review" OR "systematic review" OR "meta-analysis") |
| Mental Model Differences | ("Models, Psychological" OR "mental models") AND "Professional-Patient Relations" NOT ("review" OR "systematic review" OR "meta-analysis") |

**Table S4.** Search terms and exact strings utilized to retrieve empirical literature on patient-provider communication barriers from Current Contents Connect. The search was conducted from Jan 1 2004 to Apr 30 2026. English Journal articles were filtered in the screening process. Date filters were applied manually.

| **Scope** | **Search Terms** |
| --- | --- |
| General Search | ("communication barriers") AND ("patient-provider") NOT ("review" OR "systematic review" OR "meta-analysis") *(Topic)* |
| General Search | (("language barriers" OR "cultural barriers" OR "psychological barriers" OR "technological barriers")  AND ("healthcare" OR "clinical communication" OR "hospital interactions"))  AND ("patient outcomes" OR "diagnostic accuracy" OR "treatment adherence")  NOT ("review" OR "systematic review" OR "meta-analysis") *(Topic)* |
| Language Barriers | ("language barriers" OR "limited English proficiency" OR "non-native speakers" OR "translation issues" OR "interpreter services") AND (("Delivery of Health Care") OR "healthcare" OR "medical settings" OR "clinical communication") AND (("Treatment Outcome" OR "Patient Satisfaction") OR ("patient outcomes" OR "satisfaction")) NOT ("review" OR "systematic review" OR "meta-analysis") *(Topic)* |
| Cultural Barriers | (("Cultural Competency" OR "Health Disparities") OR ("cultural barriers" OR "cross-cultural communication" OR "ethnic disparities" OR "belief systems")) AND (("Delivery of Health Care") OR "healthcare" OR "hospital communication" OR "clinical interactions") AND (("Medication Adherence") OR "treatment adherence" OR "patient trust") NOT ("review" OR "systematic review" OR "meta-analysis") *(Topic)* |
| Psychological Barriers | (("Stress, Psychological" OR "Anxiety") OR "psychological barriers")  AND ("Professional-Patient Relations" OR "patient-provider communication")  AND ("Treatment Outcome")  NOT ("review" OR "systematic review" OR "meta-analysis") *(Topic)* |
| Technological Intervention | (("Telemedicine" OR "Health Information Technology") OR ("technological barriers" OR "digital divide")) AND (("Delivery of Health Care") OR "healthcare delivery") AND (("Patient Participation") OR "patient engagement") NOT ("review" OR "systematic review" OR "meta-analysis") *(Topic)* |
| Impacts of Communication Barrier | ("Communication" OR "Language Barrier" OR "Cultural Competency") AND "Treatment Outcome" AND "Health Services" NOT ("review" OR "systematic review" OR "meta-analysis") *(Topic)* |
| Interventions | ("Intervention Studies" OR "Patient-Centered Care")  AND ("Language Barrier" OR "Cultural Competency" OR "Stress, Psychological" OR "Telemedicine")  AND "Delivery of Health Care"  NOT ("review" OR "systematic review" OR "meta-analysis") *(Topic)* |
| Mental Model Differences | ("Models, Psychological" OR "mental models") AND "Professional-Patient Relations" NOT ("review" OR "systematic review" OR "meta-analysis") *(Topic)* |
