# Supplementary material for: Communication Barriers in Patient-Provider Interactions in Health Care: Scoping Review
Source: J Med Internet Res. 2026 Jul 21;28:e79744. doi: 10.2196/79744 (PMC13387742; doi:10.2196/79744)
Supplement: Multimedia Appendix 3 — Detailed matrix mapping specific communication barriers to their mechanisms of action and the resulting adversarial clinical outcomes. [file jmir-v28-e79744-s003.docx]

**Table S1.** Detailed matrix mapping specific communication barriers to their mechanisms of action and the resulting adversarial clinical outcomes.

| **Barrier Category** | **Specific Barrier** | **Mechanism of Action** | **Adversarial Outcome** | **Ref** |
| --- | --- | --- | --- | --- |
| Language Barriers | LEP / No Interpreter | Failure to encode symptoms or decode instructions. Exclusion from narrative. | Diagnostic inaccuracy; Medical errors; Missed red flags. | [46-48, 58-59, 65-67, 70-72, 104, 143-144] |
|  | Ad-Hoc Interpreter | Filtering or sanitizing of info by family. Lack of medical vocabulary for untrained interpreter. | Omission of critical symptoms; Breach of confidentiality. | [64, 154-160, 164-169] |
| Cultural Barriers | Belief system conflict | Conflict between religious/cultural norms and medical advice. | Non-adherence to diet/treatment; Resistance to care. | [106, 144, 172-174, 188, 200] |
|  | Non-verbal communication | Misinterpretation of body language and other non-verbal cues. | Feeling of loss of respect. The patients become more resistant to care. | [85, 91, 143, 178-179, 194] |
|  | Authority and Disclosure | Disregard for family hierarchy; Perceived disrespect. | Feeling of "Powerlessness"; Perception of racism. | [69, 86-87, 180-182, 186-187] |
| Psychological Barriers | Stigma | Fear of judgment causing concealment of history. | Withholding of sensitive information. | [60, 84, 176, 187, 214-215, 217] |
|  | Emotional overload and discomfort | Emotional distress, fear, anxiety, and low confidence | Leads to disjointed and incomplete information. | [135-137] |
|  | Psychological safety | Being intimidated in a conversation thinking that it will be dismissed. | Withdrawal from interaction. | [51, 108, 224-226] |
| Mental Model Differences | Reasoning Gap | Divergence between laymen understanding and complex medical diagnosis. | Decisional paralysis; "Talking past one another". | [143, 227, 236-239] |
|  | Assumption Divergence | Patient and provider has different preconceived assumptions. | Perception of uncaring provider. | [168, 237-239, 244] |
|  | Expectation Gap | Conflict between "Cure" (patient) vs. "Manage" (doctor). | Creates frustration and leads to feeling that provider is uncaring. | [33, 36, 194, 207, 249] |
